# The regulation of emotions: Gender differences

**DOI:** 10.1192/j.eurpsy.2021.2209

**Published:** 2021-08-13

**Authors:** I. Delhom, J.C. Melendez, E. Satorres

**Affiliations:** 1 Psychology, Valencian International University, Valencia, Spain; 2 Development Psychology, University of Valencia, Valencia, Spain; 3 Developmental Psychology, University of Valencia, Valencia, Spain

**Keywords:** women, emotion, Emotional Regulation, Gender

## Abstract

**Introduction:**

Emotional regulation, understood as the emotional ability to repair emotional states, is a skill closely linked to adaptation during aging. People who are capable to manage their emotions have greater control over moods, applying adaptive regulation strategies that allow them to maintain positive moods and modify or regulate negative ones. It has been observed that gender can be a relevant variable related to emotional regulation. In this sense, it is considered that women may be more skillful than men to emotional regulation strategies, benefiting from more successful emotional management strategies.

**Objectives:**

Verify if there are differences in the ability of emotional regulation between older men and women.

**Methods:**

The sample consisted of 851 healthy older adults, of whom 299 were men and 554 women. The participants were recruited from elderly leisure centers in the city of Valencia (Spain). To assess emotional regulation, the regulation dimension of the Trait Meta-Mood Scale 24 (TMMS-24) was used.

**Results:**

Significant differences were obtained in the emotional regulation dimension based on gender (F1, 851 = 0.075, p = 0.010), finding higher levels in women than in men (3.64 vs. 3.49).

**Conclusions:**

There is an apparent advantage of women in relation to emotional regulation, showing more skill than men in the management of emotional states. This is an important finding considering the impact of emotional regulation on adaptation during aging. This adaptative advantage has a greate importance in generation pleasant emotional states that contribute to healthy aging.

**Disclosure:**

No significant relationships.

